# Classification for Human Balance Capacity Based on Visual Stimulation under a Virtual Reality Environment

**DOI:** 10.3390/s19122738

**Published:** 2019-06-18

**Authors:** Haiyan Jin, Le Xie, Zhaolin Xiao, Ting Zhou

**Affiliations:** 1 Department of Computer Science and Engineering, Xi’an University of Technology, Xi’an 710048, China; 2171221052@stu.xaut.edu.cn (L.X.); xiaozhaolin@xaut.edu.cn (Z.X.); zhoutingmine@163.com (T.Z.); 2 Shaanxi Key Laboratory for Network Computing and Security Technology, Xi’an 710048, China

**Keywords:** balance ability classification, multi-barycentric area model, virtual reality, video analysis

## Abstract

The normal and disordered people balance ability classification is a key premise for rehabilitation training. This paper proposes a multi-barycentric area model (MBAM), which can be applied for accurate video analysis based classification. First, we have invited fifty-three subjects to wear an HTC (High Tech Computer Corporation) VIVE (Very Immersive Virtual Experience) helmet and to walk ten meters while seeing a virtual environment. The subjects’ motion behaviors are collected as our balance ability classification dataset. Secondly, we use background differential algorithm and bilateral filtering as the preprocessing to alleviate the video noise and motion blur. Inspired by the balance principle of a tumbler, we introduce a MBAM model to describe the body balancing condition by computing the gravity center of a triangle area, which is surrounded by the upper, middle and lower parts of the human body. Finally, we can obtain the projection coordinates according to the center of gravity of the triangle, and get the roadmap of the subjects by connecting those projection coordinates. In the experiments, we adopt four kinds of metrics (the MBAM, the area variance, the roadmap and the walking speed) innumerical analysis to verify the effect of the proposed method. Experimental results show that the proposed method can obtain a more accurate classification for human balance ability. The proposed research may provide potential theoretical support for the clinical diagnosis and treatment for balance dysfunction patients.

## 1. Introduction

Imbalance capacity can seriously affect people’s normal study and life. The number of disordered people in the world has increased year after year, and many of them are prone to anxiety or may even give up treatment due to the tests and training being limited, meaning they cannot achieve their desired results in a reasonable amount of time. Therefore, being able to screen out disordered people according to their balance ability, and improve their balance ability through training has an important practical significance.

At present, the existing research on human balance ability is mainly based on physical testing and mechanical testing, whose fundamental principles are based on the shift of the center of gravity, moving speed and trajectory, and other relevant parameters [1,2]. Inspired by this research, a multi-barycentric area model (MBAM) is proposed to test body balance ability in this paper. While typical people can integrate into society easily, balance disabled people may have resistance due to their physical struggles and fear. In recent years, with the rapid development of VR technology, researchers have developed some VR systems suitable for rehabilitation training, which is more safe and feasible for testing and training people with disordered suffering [3,4]. The literature [5] designed and developed a virtual dolphinarium for potential autism intervention. The autistic children will be allowed to act as dolphin trainers at the poolside and to learn (nonverbal) communication through hand gestures with the virtual dolphins, which will promote their learning and positive behavior, and can help them improve their communication, and social interaction and learning abilities. The literature [6] studied the method of relieving neural pressure based on virtual reality technology. Participants accept VR-based stress therapy, including the islands environment, forest environment, and soothing music. Aiming at a similar target, we intend to eliminate psychological pressure of disordered suffering people by improve the testing and training process.

Accordingly, we propose a classification method for those with a balance ability problem based on MBAM and hope to carry out balance ability rehabilitation training for them. The balance ability detection and rehabilitation training system is designed based on a HTC VIVE helmet VR platform. Both normal and balance disordered people use the same virtual scene while capturing their moving behavior. Then, the training videos are extracted to multiple features, such as the roadmap, moving speed, multiple gravity centers. Finally, all the features of the balance disordered people can be obtained and analyzed for classification and potential training purposes.

## 2. Methods

### 2.1. Participants

The existing literature indicates that the individual experience in a VR environment varies greatly due to age and physical condition [7,8,9,10]. The immersive virtual environment can produce a sense of dizziness and discomfort with the visual stimulation to subjects [11,12], and the feeling of discomfort for the elderly and weak is stronger. There are 53 volunteers (32 males, 21 females) from a university, aged between 18 and 27 years (mean age 24.78 ± 2.17 years, mean height 1.69m ± 0.18, mean weight 68.24kg ± 13.67) with normal indexes of physical examination in recent participant experiments. Some volunteers disclosed that they have occasionally have symptoms such as carsickness, fear of heights, and prone to falling. The experiments in this work do not involve the volunteers’ further detailed information for privacy, and each participant’s information will be replaced by a number where needed in the experiment.

### 2.2. Human Body Posture Modeling

**Figure 1:**
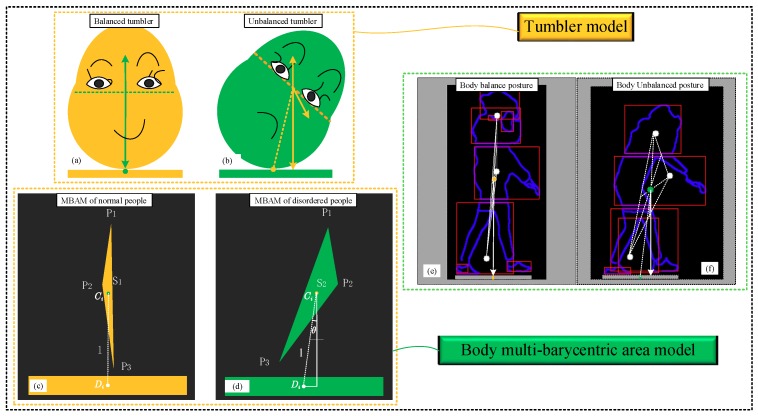
Figure of multi-barycentric area model (MBAM). (**a**) Balanced tumbler. (**b**) Unbalanced Tumbler. (**c**) MBAM of normal people. (**d**) MBAM of disordered people. (**e**) Balanced human body posture. (**f**) Unbalanced human body posture.

Many indicators and features can be measured for balance ability of the human body. In this paper, we design an area-based balanced posture model and use the balance principle of a tumbler to explain the relationship between the triangle area and the balance ability. As shown in Figure 1a, the tumbler is in a state of equilibrium, and gravity and supporting forces act in opposite directions. As shown in Figure 1b, the gravity centers will be not in a line when the tumbler is in an unbalanced state. Figure 1c shows the MBAM of the typical people, and Figure 1d is the MBAM of the balance disordered people, where *P*_1_, *P*_2_, and *P*_3_ are the center of gravities of the upper, middle and lower part of the human body, respectively; and *S*_1_ and *S*_2_ are the areas of the triangle composed of three centers of gravities. *θ* is the tilt angle of the body, which can be calculated with gravity centers. *l* is the distance between *C_i_* and *D_i_*. We choose the middle position of the two feet as the contact point *D_i_*, since the two feet will be landed alternately when people are walking.

In general, *l* is the smallest and *C_i_* is the lowest when the tumbler is in a balanced state. The line connecting *C_i_* and *D_i_*, and the vertical line from *C_i_* to the ground will be overlapping with each other. The triangle area and *θ* are also very small; at this time the body posture tends to be in a line. When the body deviates from its equilibrium state, the gravity center *C_i_* of the triangle will rise and *θ* will increase gradually, as similar to the people shown in Figure 1e,f.

The establishment and analysis of the whole model are based on the Bayesian probability theorem [13] as shown in Equation (1):

(1) P(A|B)=P(B|A)P(A)/P(B),P(A|B)∝P(B|A)P(A)

where *A* represents the normal posture of the tester, and *B* represents the triangle area composed of three barycenters.

While peoplewalking, the average area of multi-barycenters are calculated and indicated by *η*_1_ and *η*_2_ for typical people and disordered people, respectively, as shown in Equations (2)–(4).

(2) $$l=1/n\sum_{i=1}^{n}d(D_{i},C_{i})$$

(3) $$\eta_{1}=1/n\sum_{i=1}^{n}\left(\left|d(D_{i},C_{i})-l\right|\sum_{i=1}^{n}\left(S_{\left(p_{1},p_{2},p_{3}\right)}/n\right)\right)$$

(4) $$\eta_{2}=1/n\sum_{i=1}^{n}\left(\left|d^{\prime }(D^{\prime }{}_{i},C^{\prime }{}_{i})-l\right|\sum_{i=1}^{n}\left(S^{\prime }{}_{\left(p_{1},p_{2},p_{3}\right)}/n\right)\right)$$

where *n* is the number of the subjects. *D_i_* and *D*′*_i_* are the middle position between two feet of each step for the *i*-th normal and the *i*-th disordered. *C_i_* and *C*’*_i_* are the gravity center of the triangle composed of multi-barycenters of the *i*-th normal and the disordered. *d*(*D_i_*,*C_i_*) represents the distance between *D_i_* and *C_i_*, and *l* represents the mean distance between *C_i_* and *D_i_* for *n* subjects. *p*_1_, *p*_2_, and *p*_3_ are three gravity centers of each person, and *S* (*p*_1_, *p*_2_, *p*_3_) and *S*′ (*p*_1_, *p*_2_, *p*_3_) are the area of triangle that can be used as an effective metric for the balanced and unbalanced status.

If the area of multi-barycenter is less than *η*_1_, it can be identified as the normal. Otherwise, it will be identified as disordered if it is greater than *η*_2_. However, it is more difficult to judge if it is between *η*_1_ and *η*_2_, and this could be determined roughly by setting a threshold *T* according to the experience. However, the level of accuracy is not very high, and it needs to be combined with other strategies.

In addition, in the experiments we also introduce the variance indicated by *σ*^2^ defined as Equation (5).

(5) $$\sigma^{2}=\sum_{i=1}^{n}\left({\left(S_{i}^{t}-\bar{S_{i}}\right)}^{2}/n\right)$$

where $S_{i}^{t}$ is the area of the triangle at a certain time *t*, and $\bar{S_{i}}$ is the average area of all time during the walking for the *i*-th person.

### 2.3. Human Body Posture Feature Extraction and Classification

The feature extraction and classification process of unbalanced body posture consists of three steps: Firstly, differential and filtering are performed as image preprocessing on the video data; Then, the based balance ability classification is achieved by using the support vector machine (SVM) method after thefeature extraction; Finally, the experiments are conducted on real datasetsto verify the proposed classification.

**Figure 2:**
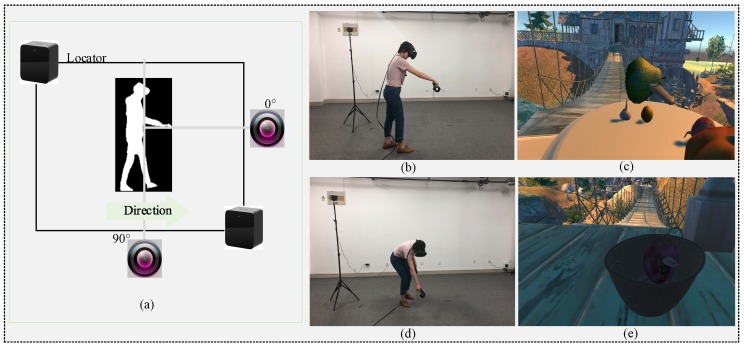
The subject walking in theVR scene. (**a**) The chart of experiment scene scheme. (**b**) The action of the subject for picking up a piece of fruit in the real environment. (**c**) VR scene corresponding to (**b**). (**d**) The action of the subject for putting down a piece of fruit in the real environment. (**e**) VR scene corresponding to (**d**).

**Figure 3:**
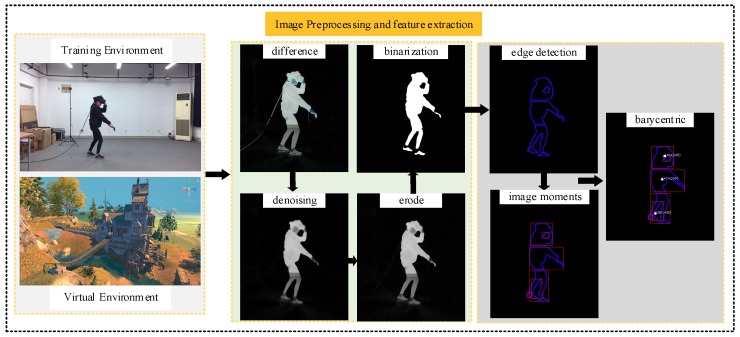
The whole process of image preprocessing.

The process of image preprocessing and feature extraction is described in Figure 2 and Figure 3. As shown in Figure 2a, the system can locate the positions of the helmet and handle with the HTC VIVE platform. The training videos are taken from the front and side of the walking path. The subject gets the designated fruit or vegetable from the table at the bridgehead (Figure 2b) by following the instructions. Then, they are required to place the objects into the basket at the bridge-end, as seen in Figure 2d–e. In the real experimental environment, in order to facilitate the following image processing, we request the subjects wear dark clothes as the wall is white.

In order to summarize the proposed model, our preprocessing process can be summarized by the following steps as shown in Figure 3.

Step 1. Video frame preprocessing. Among the obtained experimental video data, taking one frame from each consecutive two frames and save it for further processing, named *I_p_*.
Step 2. Difference algorithm. The image *I_p_* and blank scene *I_e_* are used to make a difference to obtain the difference image *I_dif_* [14], as shown in Equation (6).
  (6) $$I_{dif}=I_{p}-I_{e}$$
Step 3. Image denoising. We use a bilateral filter to denoise [15].
Step 4. Using the edge detection operator to process the corroded grayscale image to obtain the connected region of the image [16].
Step 5. The image moments are used to describe feature parameters. An image is a two-dimensional plane, and the pixel value of each point can be regarded as the density of the point. The expectation of that point is the moment of it.

As shown in Equation (7), if it is a binary image, *V*_(*i*,*j*)_ has only two values: 0 (black) and 1 (white).

(7) $$M_{10}=\sum_{i}\sum_{j}i\cdot V(i,j),M_{01}=\sum_{i}\sum_{j}j\cdot V(i,j),M_{00}=\sum_{i}\sum_{j}V(i,j)$$

where *i* and *j* are the coordinate of the point, respectively, and *M*_10_ and *M*_01_ are the accumulation of the *x* coordinate and *y* coordinate of all white areas of the image, respectively. Therefore, (*x_i_*, *y_i_*) is the barycentric coordinate of the image calculating by Equation (8). Accordingly, the triangle area can be obtained according to the upper, middle and lower center of gravities [17].

(8) $$x_{i}=M_{10}/M_{00},y_{i}=M_{01}/M_{00}$$

The pseudocode for extracting features is as follows:
**Feature extraction pseudocode**1: Begin Function 2: Obtain training videos 3: Video image preprocessing. Take one frame from each two consecutive frames and record. 4: Select pure background image without person as *I_e_*.          Select images with person walking as *I_p_*. 5: Do image difference (*I_dif_* = *I_p_* − *I_e_*). 6: Image denoising using bilateral filtering 7: Search body silhouette using edge detection operator 8: Calculate the smallest rectangle that is perpendicular to the boundary of body silhouette 9: Search the upper, middle and lower barycenter of the human bodyaccording to the image moment, (*center*_1_, *center*_2_, *center*_3_) 10: Calculate the area of a triangle composed of three centers of multiple barycenters. 11: Draw a walking roadmap according to the projection of the triangle. 12: Calculate walking speed of the first half and the second half during the whole walking time, respectively. 13: End function

### 2.4. SVM-Based Balance Ability Classification

SVM (Support Vector Machine) is a very effective and practical method for solving binary classification problems, which maps input vectors to high-dimensional feature spaces through pre-selected nonlinear mapping relationand constructs a linear classification in the feature space, and determines the final decision function by solving the dual problem [18].

In this paper, the triangle area composed of multiple barycentres is regarded as input data of SVM, and the classification accuracy is 87.8788%. We take one frame from each consecutive two frames, and the data selected follows a Gaussian distribution. For example, in all recorded image frames, 5% of the images are extracted in the first 20% of the time period, and 40% in the middle 60% of the time period, and 5% in the last 20% of the time period for analysis. Table 1 shows the correct label and the test label of the test data for 24 groups, where 1 indicates the label of the normal people and −1 indicates the label of the disordered people. According to the classification result of Table 1, only one group of classification result is wrong, which verifies the validity of the MBAM model.

## 3. Results

### 3.1. Analysis of Experimental Results

At present, the assessment of human body balance ability mainly includes traditional observation, scale evaluation and balancing instruments [19,20]. Also, more and more VR-based techniques are used in clinical assessment of balance ability [21]. In this article, we apply classification method based on visual stimulation for human balance ability in VR environment, which is safe, easy to operate, and low cost. This method can acquire the data of the multi-barycentric area during walking and classify accordingly. We expect this system could do some meaningful work for further rehabilitation training for people with balance dysfunction.

Table 2 is the comparison of classification accuracy for body balance ability in 12 normal subjects and 12 balance dysfunction subjects in VR environment and physical environment, respectively. We select 300 frames of images for each participant to analyze. The classification accuracy of balance ability for normal people both in VR environment and physical environment is 100%. However, the classification results for the disordered in VR environment and physical environment are quite different, and that is about 91.67% and 58.33%, respectively. In a VR environment, subject No.23 missed detection, and in a physical environment, subjects No.13, 15, 18, 23 and 24 missed detection. Analyzing the reasons: In the actual physical environment, subjects can see their body and the real circumstances, so they can rely on the information obtained by their eyes to predict the comingmotion and make posture adjustments in advance. Compared with physical scenes, in the VR environment (with a helmet), subjects cannot see their body and the whole real physical scene; therefore, they could not predict the next action, and adjust their body or make any preparation in advance. That is to say, visual correction effectsin advance can be eliminated partly in a VR environment, and thus thebalance dysfunctioncan be detected more objectively. Hence, for disordered people in a VR environment, the detection accuracy is much higher than in a physical environment.

#### 3.1.1. Analysis of the Multi-Barycentric Area

**Figure 4:**
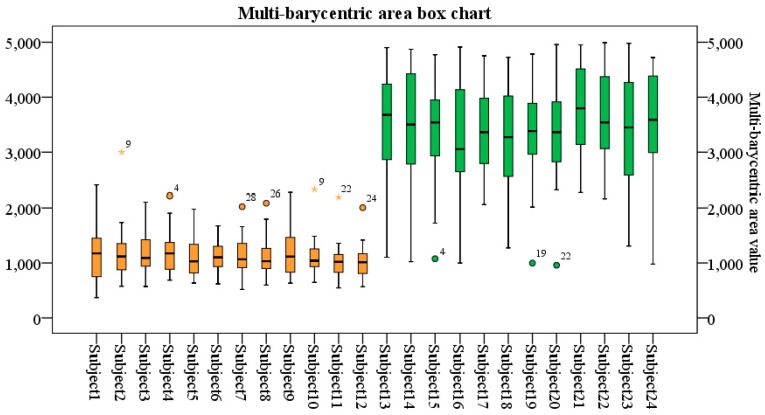
Comparison of multi-barycentric areas.

Among 24 subjects, 30 groups of data are selected for each subject according to the Gaussian distribution mentioned in Section 2.4 for analysis. As shown in Figure 4, subjects 1 to 12 are normal, and the area data are distributed between 300 and 2000; Subjects 13 to 24 with poor balance ability, the area data are distributed between 2200 and 6000. Virtual scenes can easily to cause dizziness, and people with balance impairment are more likely to be affected. Experimental data prove that the triangle area enclosed by the multi-barycentric of the normal is smaller than that of people with balance disorders in the VR scene.

#### 3.1.2. Analysis of Variance of the Multi-Barycentric Area

**Figure 5:**
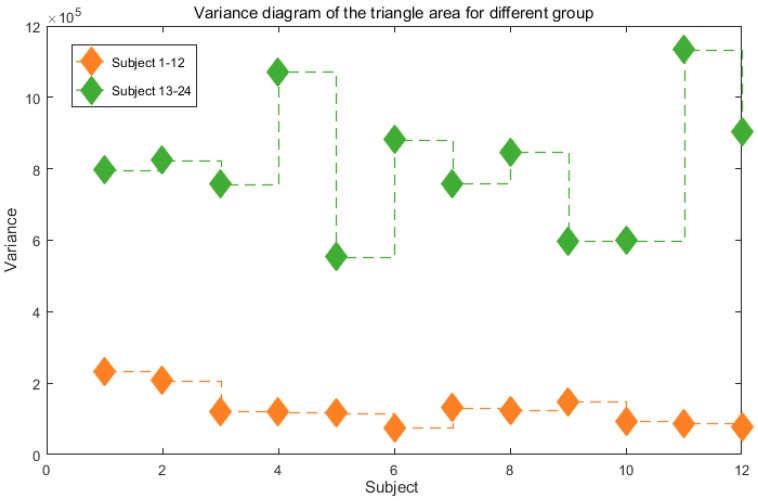
Comparison of multi-barycentric area for different people.

In the experiment, 12 typical people and 12 people with balance impairment are recorded. The multi-barycentric area composed of the upper, middle and lower of human body is obtained by pretreatment process mentioned in image preprocessing and feature extraction. The area variance is shown in Figure 5. The experimental results show that the variance of multi-barycentric area in typical people is small, and the body posture shaking is not obvious compared with that in people with a balance disorder.

#### 3.1.3. Analysis of Roadmap and Moving Speed

Because of the visual stimulation of the virtual scene, the subject may appear dizzy when walking [22,23]. In the experiment, fivetypical subjects and five disordered subjects from 53 subjects are selected randomly.

**Figure 6:**
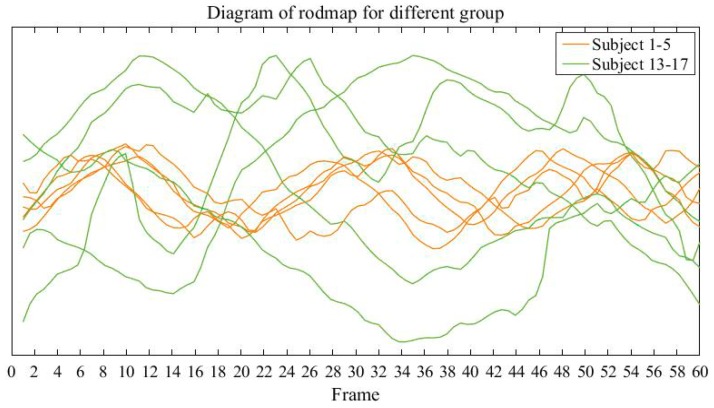
Comparison of the roadmap for different groups.

For each person, we select 60 sets of data computed by the triangular projection coordinates composed of multiple barycentres, and then connect the projection coordinates sequentially to get the roadmap of each person. As shown in Figure 6, orange lines correspond to the walking track of five typical subjects and the green lines correspond to five disordered subjects. From the roadmap, compared with the typical subjects, we can see that the range of body moving of disordered subjects is much larger than that of the typical subjects during the walking. Therefore, we can judge the balance ability of subjects preliminarily by analyzing their walking routes roughly.

**Figure 7:**
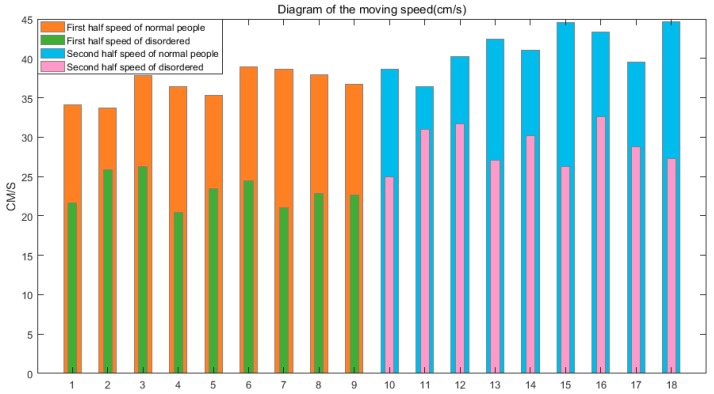
Comparison of the moving speed for different groups (cm/s).

Similarly, we can obtain the walking speed of 53 subjects. When they enter the virtual reality scene in the early stage, they felt dizzy and walk slowly. After a period of training, this phenomenon gradually disappeared. As shown in Figure 7, the walking speed of nine typical people and nine balance impaired people in the first half and the second half in virtual reality environment are calculated, respectively. We find that the walking speed of normal people was basically faster than that of balance impaired people.

## 4. Discussion

A MBAM model based on video analysis to assess the balance ability of the human body is proposed in the article. After analyzing the training video data, we find that there is a significant difference in the balance ability between typical and balance disordered people. The triangle area and area variance of the typical people during walking are smaller than that of the disordered. By the comparison of roadmap and walking speed, compared with people with balance impairment, the body shaking of typical people in the virtual environment is not obvious, and the moving speed is faster. Subjects walking in the VR environment for a long time will have a visual adaptation process. During the visual adaptation process, the balance ability score increases as training time increases. Our method can effectively be used to classify balance ability, and we hope it will provide clinical diagnosis and theoretical support for the treatment of patients with balance dysfunction in clinical medicine.

The proposed model has a good classification on human balance ability, but we realize that there are two limitations in the current study. Firstly, the subjects are all the adult young people aged between 18 and 27 years, and there is no strong diversity at the age level of the subjects. Secondly, the sample size in the experiments is 53, and it is insufficient. Even so, this original MBAM research has initially shown its effectiveness in the experiments. In the following study, we plan to carry out the research from two aspects. One is to introduce a deep learning frame to find better features for human balance capacity classification. The other is to select subjects of different ages and levels to increase the sample diversity in experiments to verify the effectiveness of more methods.

## Figures and Tables

**Table 1:** Classification test data and labels.

**Data**	387	326	382	297	362	351	310	390	281	353	376	389
**Correct label**	1	1	1	1	1	1	1	1	1	1	1	1
**Test label**	1	1	1	1	1	1	1	1	1	1	1	1
**Data**	378	325	355	382	279	267	372	254	268	367	322	371
**Correct label**	−1	−1	−1	−1	−1	−1	−1	−1	−1	−1	−1	−1
**Test label**	−1	−1	−1	−1	−1	−1	−1	−1	−1	−1	1	−1

**Table 2:** Comparison of classification accuracy under a VR environment and a physical environment.

Subject Category	Experimental Environment	The Number of Subject	Correct Classification	Accuracy
**The normal (Subject No.1~12)**	VR environment	12	12	100%
	Physical environment	12	12	100%
**The disordered (Subject No.13~24)**	VR environment	12	11	91.67%
	Physical environment	12	7	58.33%
